# Solid type primary intraosseous squamous cell carcinoma in the maxilla: report of a new case

**DOI:** 10.1186/1472-6815-13-13

**Published:** 2013-12-31

**Authors:** Mitsuyoshi Iino, Shigeo Ishikawa, Hisashi Ozaki, Takehito Kobayashi, Hirohiko Tachibana, Hiromasa Sakurai, Noriaki Kikuchi

**Affiliations:** 1 Division of Dentistry, Oral and Maxillofacial Surgery, Faculty of Medicine, Yamagata University, 2-2-2 Iida Nishi, Yamagata City Yamagata 990-9585, Japan; 2 Division of Plastic and Reconstructive Surgery, Faculty of Medicine, Yamagata University, 2-2-2 Iida Nishi, Yamagata City Yamagata 990-9585, Japan

**Keywords:** Primary intraosseous squamous cell carcinoma, Solid type, Maxilla

## Abstract

**Background:**

Primary intraosseous squamous cell carcinoma is a rare jaw bone tumor defined as squamous cell carcinoma arising within the jaw, having no initial connection with the oral mucosa and developing from residual odontogenic epithelium or from an odontogenic cyst or tumor. Solid type of this tumor arising in the maxilla is an even rarer presentation, because the majorities derive from cystic lesions and are found in the posterior mandible.

**Case presentation:**

A 36-year-old Japanese man was referred to our clinic with a complaint of pain around the anterior maxillary region on the right side. Intraoral examination identified a firm, non-fluctuant mass with no ulceration in the vestibular region of teeth #11-13. Incisional biopsy was performed, leading to histological diagnosis of moderately differentiated squamous cell carcinoma. Contrast-enhanced computed tomography revealed a destructive tumor with no cystic lesion, >50 mm in diameter. There was no evidence of metastatic disease on chest radiography, upper gastric endoscopy or positron emission tomography. Based on these findings, solid type primary intraosseous squamous cell carcinoma was diagnosed. The patient underwent tumor ablative surgery. The surgical defect was reconstructed using a partially double-folded free radial forearm flap and prefabricated denture-based surgical obturator. The postoperative course was quite good. Neither recurrence nor metastasis had been found as of 3 years and 1 month postoperatively.

**Conclusion:**

To the best of our knowledge, the present case represents only the 10th case of solid type primary intraosseous squamous cell carcinoma arising in the maxilla to be reported.

## Background

Primary intraosseous squamous cell carcinoma (PIOSCC) is a rare jaw bone tumor defined as a squamous cell carcinoma (SCC) arising within the jaw, having no initial connection with the oral mucosa, and, presumably, developing from residual odontogenic epithelium or from an odontogenic cyst or tumor [1]. PIOSCC is classified into solid type, derived from keratocystic odontogenic tumor, and derived from odontogenic cysts [1]. The majority of PIOSCCs are found in the posterior mandible and derived from a cystic lesion [2-7]. Solid type PIOSCC arising in the maxilla is thus an even rarer presentation [2-7]. We present herein a new case of successfully treated solid type PIOSCC of the anterior maxilla.

## Case presentation

**Figure 1:**
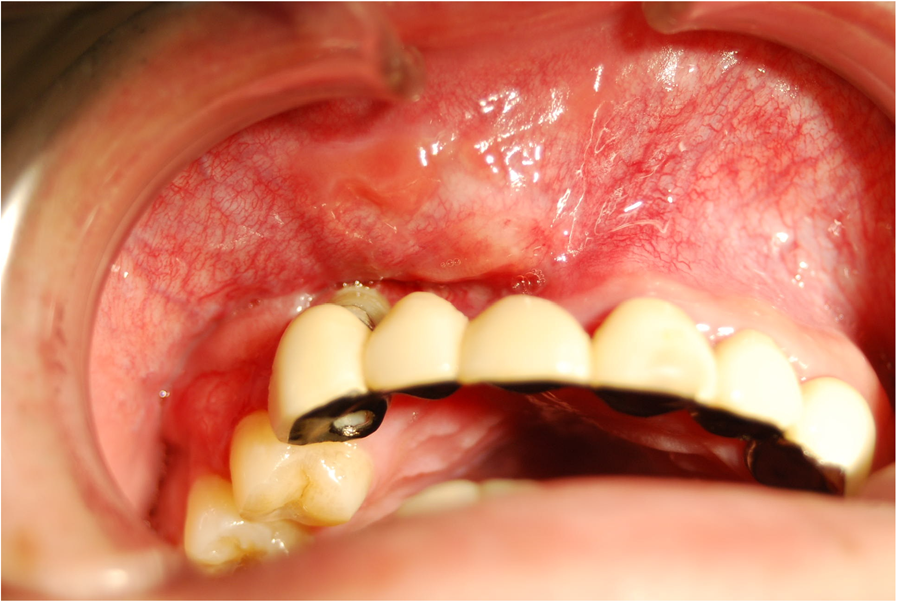
Intra-oral view at first visit, showing a firm and non-fluctuant submucosal swelling of the anterior maxilla.

**Figure 2:**
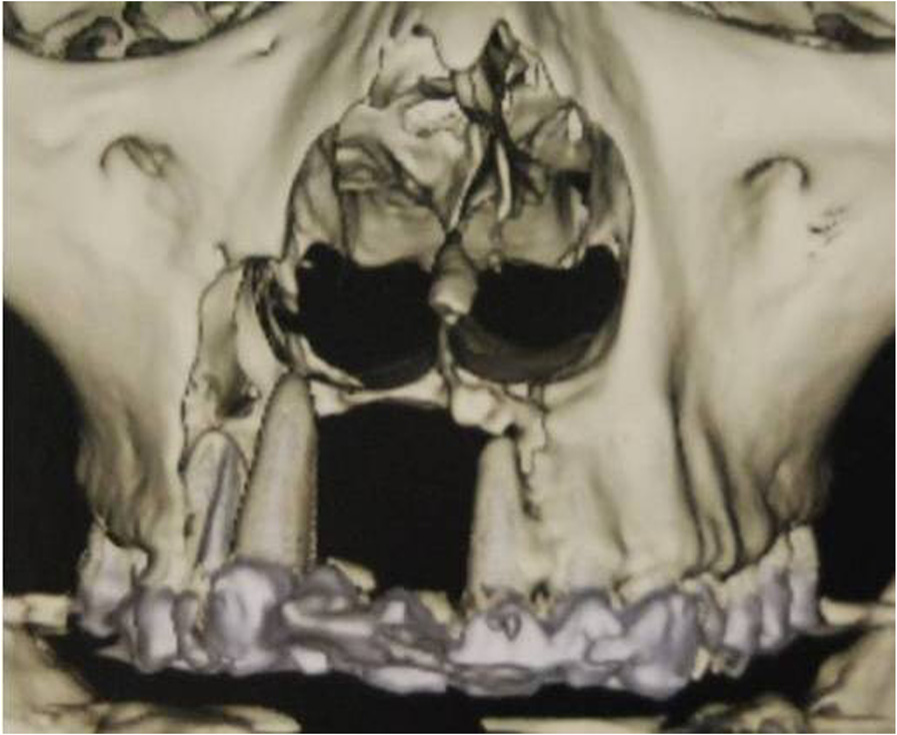
3D-CT showing severe bony resorption between the right upper second premolar and the left upper lateral incisor.

**Figure 3:**
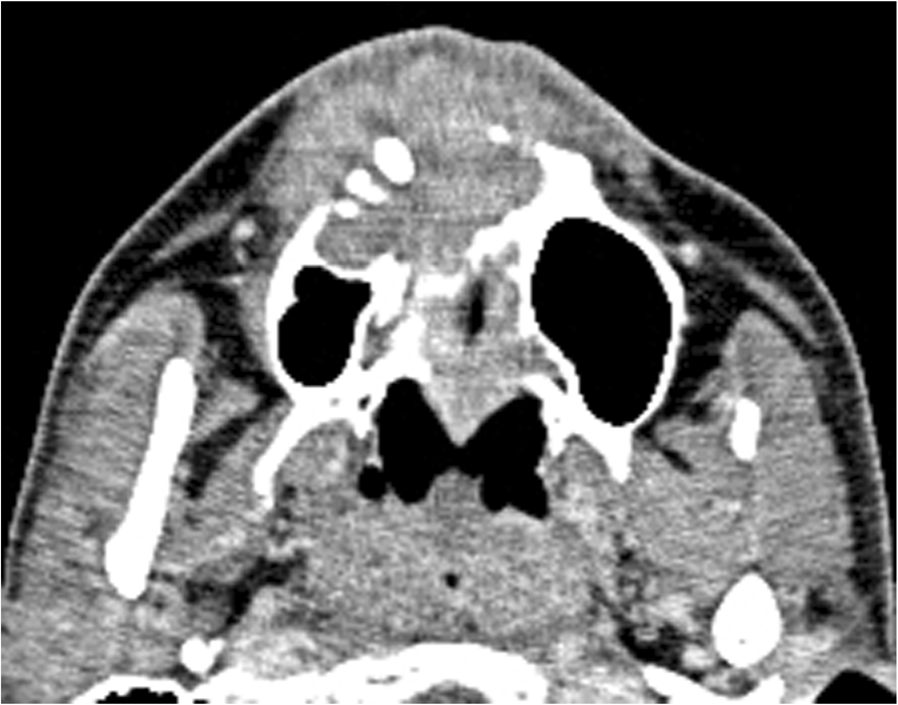
Preoperative CE-CT showing a large, destructive tumor located in the maxilla.

A 36-year-old man was referred to our clinic from his family dentist with a complaint of pain around the anterior maxillary region on the right side. The patient’s medical history was non-contributory. Intraoral examination identified a firm, non-fluctuant mass with no ulceration in the vestibular region between the right upper central incisor and canine (Figure 1). The right upper central and lateral incisors were missing. A computed tomography (CT) showed severe bony resorption between the right upper second premolar and the left upper lateral incisor (Figure 2). Incisional biopsy was performed via vestibular approach. The specimen of the tumor was extirpated with overlying mucosa. During the biopsy, an intact cortex was not observed. The histological diagnosis was moderately differentiated SCC. The connection between the tumor and the overlying mucosa was not observed. Contrast-enhanced computed tomography (CE-CT) revealed a destructive tumor with no cystic lesion, >50 mm in diameter. The tumor pressed the skin around the nasal ala and the nostril of the right side, but no direct connection between the skin and tumor was observed. Although the maxillary sinus and nasal cavity were deformed by the tumor, no abnormal findings were seen for the mucosa of the nasal cavity and maxillary sinus (Figure 3). One swollen and enhanced lymph node was recognized in the submandibular area of the right side. Positron emission tomography (PET) revealed the enhanced lesion in the maxilla and the right submandibular area. There was no evidence of metastatic disease on chest radiography, upper gastric endoscopy or PET. Based on these findings, solid type PIOSCC was diagnosed.

**Figure 4:**
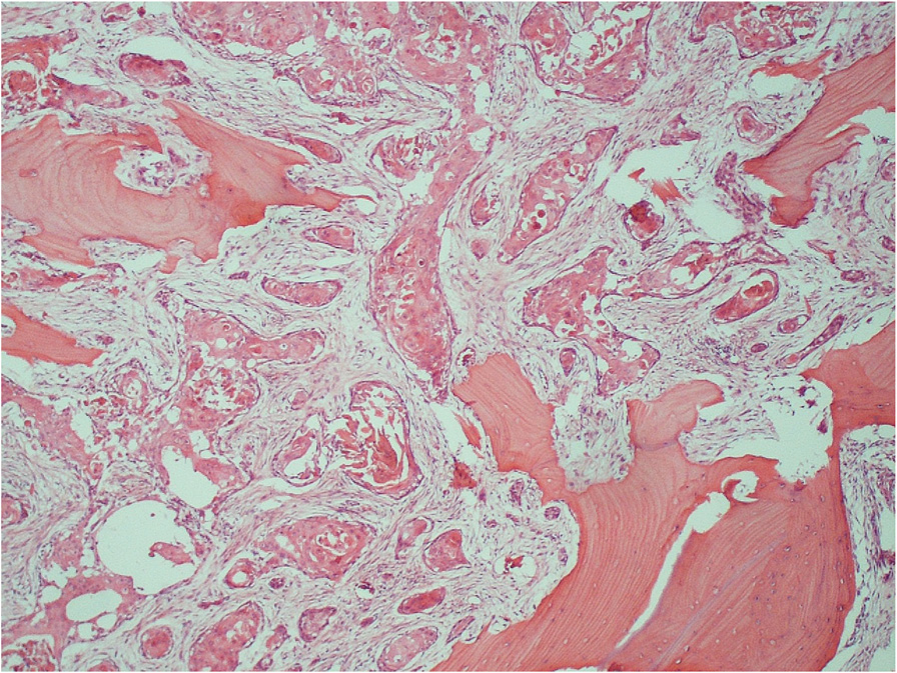
**Photomicrograph of the resected maxilla showing the moderately differentiated SCC.** The islands of the tumor cells extended into the maxillary bone.

The patient underwent tumor ablative surgery. After bilateral supraomohyoid neck dissection, the primary tumor was radically excised. The surgical specimen comprised the anterior two-thirds of the hard palate and nasal septum, bilateral inferior nasal conchae and the skin around the nasal ala and nostril of the right side. The surgical defect was reconstructed using a partially double-folded free radial forearm flap and prefabricated denture-based surgical obturator. Microscopic examination of the surgical specimen revealed SCC without cystic component in the maxillary bone. The islands of the tumor cells extended into the bone (Figure 4) with no dysplasia or carcinoma in the skin and mucosa of the nasal cavity and maxillary sinus. The dissected surgical specimen of the neck showed one lymph node metastasis of the ipsilateral submandibular region. Although adjuvant radiotherapy was recommended, the patient declined additional treatment. The postoperative course was quite good. Neither recurrence nor metastasis had been found as of 3 years and 1 month postoperatively.

## Conclusions

The diagnostic criteria for PIOSCC include absence of an initial connection with the overlying mucosa or skin and exclusion of metastasis from a distant primary tumor by physical and radiographic examination during a follow-up period of at least 6 months [2]. As the present case fulfilled all the criteria listed above and contained no cystic component, this tumor represented solid type PIOSCC in the maxilla. This tumor is thought to arise by direct malignant transformation of odontogenic epithelial rests such as remnants of dental lamina, and reduced enamel epithelium surrounding unerupted/impacted teeth [8]. In this case, the teeth #11 and 12 were removed before the first visit. Therefore, some aberrant epithelial tissue including the rests of Malassez is also the possible origin.

In a review of 200 cases reported up to 2010, Nomura et al. [7] reported only eight cases (4%) of solid type PIOSCC in the maxilla. In 2011, Mohyuddin and Yao [9] reported a new case of this tumor. Thus, to the best of our knowledge, the present case represents only the 10th case of solid type PIOSCC arising in the maxilla to be reported.

Standard treatment for PIOSCC includes surgery, radiotherapy, or both, and the prognosis is relatively poor [2-7]. All reports strongly suggest that wide resection of the primary tumor with a sufficient safety margin [2-9]. However, no standard indications or protocols for radio- or chemoradiotherapy have been established. Additional study is needed to identify optimal treatment modalities.

### Consent

Written informed consent was obtained from the patient for publication of this case report and any accompanying images. A copy of the written consent is available for review by the Editor-in-Chief of this journal.

## Competing interests

The authors declare that they have no competing interests.

## Authors’ contributions

SI and NK operated and monitored the patient. TK, MI, HO, HT, and HS assisted in the surgery and monitoring the patient. All authors read and approved the final manuscript.

## Pre-publication history

The pre-publication history for this paper can be accessed here:

http://www.biomedcentral.com/1472-6815/13/13/prepub
